# Mass distribution profiling of cell wall matrix polysaccharide biosynthesis mutants reveals alterations in non-targeted matrix polysaccharides

**DOI:** 10.1016/j.tcsw.2026.100181

**Published:** 2026-09-05

**Authors:** Supanut Utthiya, Sukhita Sathitnaitham, Anongpat Suttangkakul, Passorn Wonnapinij, Seth J. Davis, Leonardo D. Gomez, Supachai Vuttipongchaikij

**Affiliations:** a Department of Genetics, Faculty of Science, Kasetsart University, 50 Ngam Wong Wan, Chatuchak, Bangkok 10900, Thailand; b Department of Biology, University of York, Heslington, York YO10 5YW, United Kingdom; c Centre for Novel Agricultural Products (CNAP), Department of Biology, University of York, Heslington, York YO10 5DD, United Kingdom; d National Center for Agricultural Biotechnology, Kasetsart University Institute for Advanced Studies, Kasetsart University, Chatuchak, Bangkok 10900, Thailand

**Keywords:** *Arabidopsis thaliana*, Glycan-directed monoclonal antibody, Hemicellulose, Pectin, Plant cell wall, Size-exclusion chromatography

## Abstract

Plant cell walls contain multiple matrix polysaccharides whose interactions help determine wall mechanics, growth, and integrity, but it remains unclear how disruption of one biosynthetic or modification pathway reshapes other, non-target polymers. We used gel permeation chromatography coupled with enzyme-linked immunosorbent assay (GPC-ELISA) to compare mass distributions of major matrix polysaccharides in *Arabidopsis thaliana* mutants affecting xylan biosynthesis and decoration (*irx14L, irx15, gux1-1,* and *gux2-2*), glucomannan synthesis (*csla2-1*), pectin homogalacturonan (HG) biosynthesis (*gaut10-2* and *gaut11-2*), and wall esterification (*axy4-4, tbl31, tbl2, tbl3,* and *tbr*). Three biological replicates per genotype, each from independent cell wall extractions, were profiled. Disruption of a single pathway frequently altered the distribution of non-target polymers. Xylan-related mutants altered not only glucuronoxylan but also HG and, in some cases, xyloglucan, with *gux1-1* and *gux2-2* producing particularly contrasting GX and HG profiles. By contrast, *csla2-1* showed relatively limited secondary effects, whereas *gaut10-2* and *gaut11-2* showed increased low-molecular-size unesterified HG together with localised redistribution of several hemicellulose epitopes. Esterification mutants separated into distinct redistribution patterns rather than a single shared phenotype. Together, these findings support a directional and asymmetric model of cross-polymer coupling within extractable wall fractions, in which perturbation of HG or GX pathways has broader effects on the wall matrix than disruption of mannan biosynthesis.

## 1 Introduction

Plant cell walls are essential extracellular matrices that provide mechanical support, shape cell form, mediate intercellular interactions, and contribute to defense against pathogens (Cosgrove, 2023). Their composition and architecture are dynamically remodelled throughout development and in response to growth signals, environmental cues, and biotic stress (Burton, Gidley, & Fincher, 2010; Gigli-Bisceglia, Engelsdorf, & Hamann, 2019). Understanding how wall composition is established and maintained is therefore central to plant biology, with direct relevance to crop improvement, biomass utilisation, and the engineering of novel materials.

The primary cell wall of eudicots such as *A. thaliana* is organised around a framework of cellulose microfibrils embedded in a matrix of non-cellulosic polysaccharides (Bidhendi & Geitmann, 2016; Zhang, Yu, & Cosgrove, 2025). This matrix comprises two major classes: hemicelluloses and pectins. Among the hemicelluloses, xyloglucan (XyG) is the most abundant in eudicot primary walls, consisting of a β-1,4-glucan backbone decorated with xylosyl, galactosyl, and fucosyl side chains that modulate its interaction with cellulose microfibrils (Cavalier et al., 2008; Xiao, Zhang, Zheng, Cosgrove, & Anderson, 2016). Glucuronoxylan (GX), a β-1,4-xylan backbone substituted with (methyl)glucuronic acid residues, is the most abundant hemicellulose of secondary cell walls and is also present in primary walls (Anders, Wilson, Sorieul, Nikolovski, & Dupree, 2023; Brown, Zhang, Stephens, Dupree, & Turner, 2009). Heteromannan (HM), synthesised by CELLULOSE SYNTHASE-LIKE A family glycosyltransferases, contributes to mannan or glucomannan-type backbones present in both primary and secondary walls (Yu et al., 2022). The pectic polysaccharides, principally homogalacturonan (HG), rhamnogalacturonan-I (RG-I), and rhamnogalacturonan-II (RG-II), form a gel-like matrix that is particularly abundant in the primary wall and middle lamella (Mohnen, 2008). HG, the most abundant pectin, is a linear homopolymer of α-1,4-linked galacturonic acid that is secreted in a highly methyl-esterified form and subsequently de-esterified by pectin methylesterases in the apoplast, a process that can promote Ca^2^^+^−mediated crosslinking and influence wall stiffness. The degree and pattern of HG methylesterification are key determinants of wall mechanical properties and cell adhesion (Du, Anderson, & Xiao, 2022; Levesque-Tremblay, Pelloux, Braybrook, & Müller, 2015; Willats et al., 2001).

A defining feature of matrix polysaccharide biosynthesis is that, unlike cellulose synthesis at the plasma membrane, it occurs within the endomembrane system, particularly the Golgi apparatus (Driouich et al., 2012). Golgi-resident glycosyltransferases assemble the backbones of non-cellulosic wall polysaccharides and add side-chain decorations, while modification enzymes introduce acetyl and methyl groups within the Golgi lumen (Temple et al., 2022; Zhong, Zhou, Chen, & Ye, 2024). Because the biosynthetic machinery for GX, HG, XyG, and HM co-resides in the same compartment, the Golgi should be viewed not simply as a conduit for separate polymer streams, but as a shared biosynthetic hub in which coordination among pathways is likely to influence wall composition (Amos & Mohnen, 2019; Parsons et al., 2012).

Despite the structural and functional interdependence of wall polysaccharides, most genetic and biochemical studies have examined individual biosynthetic pathways in isolation. Mutations affecting GX biosynthesis, HG synthesis, HM synthesis, or wall acetylation each produce characteristic defects in their target polymers (Brown et al., 2011; Gille & Pauly, 2012; Goubet et al., 2009; Lee, Teng, Huang, Zhong, & Ye, 2010; Wu et al., 2010; Yoshimi et al., 2025; Yuan, Teng, Zhong, & Ye, 2016). However, increasing evidence indicates that perturbation of one pathway can also alter other wall components. For example, loss of XyG in *xxt1 xxt2* leads to compensatory increases in HG and glucomannan (Sowinski, Westman, Redmond, Cosgrove, & Anderson, 2022), xylan-related defects can alter the distribution of pectic polymers between wall fractions (Bromley et al., 2013), and changes in wall mechanics caused by XyG deficiency are associated with alteration in pectin composition, suggesting a compensatory role of pectin (Daher, Serra, Carter, & Braybrook, 2024). These observations suggest interdependence among wall polymers, but the extent, directionality, and mechanistic basis of these effects have not been systematically compared across multiple polysaccharide classes and modification states.

A key methodological challenge in studying cross-polymer effects is that conventional bulk compositional analyses, such as monosaccharide profiling, linkage analysis, or single-epitope immunolabelling, report total polysaccharide amounts but do not capture changes in polymer size distribution or distribution between extracted wall fractions that may accompany compensatory remodelling. Gel permeation chromatography separates polymers by molecular size, providing a molecular weight distribution profile that reflects both chain length and the degree of polymer aggregation or cross-linking (Striegel & Timpa, 1996; White Jr., 1999). GPC-ELISA has recently been shown to enable simultaneous, quantitative profiling of the molecular weight distributions of multiple specific polysaccharides from the same fractionated wall extract (Sathitnaitham et al., 2021). The use of sequential CDTA and NaOH extractions prior to GPC fractionation further allows discrimination between the chelator-soluble pectin-enriched fraction and the alkali-soluble hemicellulose-enriched fraction, providing information on the wall-binding state of each polysaccharide class.

In this study, we applied GPC-ELISA to a panel of well-characterised *A. thaliana* cell wall biosynthesis and modification mutants spanning four polysaccharide pathways: xylan backbone synthesis and decoration (*irx14L*, *irx15*, *gux1-1*, and *gux2-2*), glucomannan synthesis (*csla2-1*), pectin HG synthesis (*gaut10-2* and *gaut11-2*), and polysaccharide *O*-acetylation and esterification (*axy4-4*, *tbl31*, *tbl2*, *tbl3*, and *tbr*) (Table 1). By using previously characterised mutants across multiple pathways, we asked how disruption of one matrix polysaccharide influences the fraction-associated abundance and molecular size distribution of other non-target polymers, and extended this comparative framework beyond backbone biosynthesis to polysaccharide substitution and esterification. Our aims were to characterise the primary effects of each mutation on the molecular size distribution of its target polysaccharide, to document secondary effects on non-targeted polysaccharides, and to use these redistribution patterns to infer how matrix polysaccharide pathways are functionally linked. The results reveal directional patterns of cross-polymer effects and support a model in which shared biosynthetic coordination and post-secretory wall reorganisation together contribute to matrix polysaccharide interdependence.

**Table 1:** List of T-DNA insertion mutants used in this study.

Group	mutant	AGI locus	Accession number	References
Xylan	*irx14L*	At5g67230	SALK_066961	Brown et al., 2007
	*irx15*	At3g50220	GK-735E12	Jensen et al., 2011
	*gux1-1*	At3g18660	SALK_046841	Mortimer et al., 2010
	*gux2-2*	At4g33330	SAIL_514_E10	Lee et al., 2012a
Mannan	*csla2-1*	At5g22740	SALK_006803	Goubet et al., 2009
Pectin	*gaut10-2*	At2g20810	SALK_082273	Caffall et al., 2009
	*gaut11-2*	At1g18580	SALK_148781	Caffall et al., 2009
Esterification	*axy4-4*	At1g70230	SALK_070873	Gille et al., 2011
	*tbl2*	At1g60790	SALK_020184	Bischoff, Selbig, & Scheible, 2010
	*tbl3*	At5g01360	SALK_065959	Yuan et al., 2016
	*tbl31*	At1g73140	SAIL_612_A03	Yuan et al., 2016
	*tbr*	At5g06700	SALK_058509	Bischoff et al., 2010

## 2 Materials and methods

### 2.1 Plant materials and growth conditions

*A. thaliana* T-DNA insertion lines were obtained from the Nottingham Arabidopsis Stock Centre (NASC). Seeds of mutant lines and wild-type (Col-0) were surface-sterilized and stratified at 4 °C in the dark for 2–3 days, then germinated on half-strength Murashige and Skoog (MS) medium solidified with 1% (w/v) agar and supplemented with 1% (w/v) sucrose for 7 days. Seedlings were grown in compost at 22 °C under a 16 h light/8 h dark photoperiod. All genotypes within a mutant group were grown simultaneously under identical conditions in the same growth room using the same batch of compost and watering regime. For leaf material, all rosette leaves were harvested from 3-week-old, pre-bolting plants and pooled per biological replicate. For inflorescence stem material, the primary bolt was harvested from 5-week-old plants, and the mature and thick-walled lower portion of the stem were pooled per biological replicate, avoiding the young, still-elongating apical region. Three biological replicates were prepared per genotype, each from an independent alcohol-insoluble residue **(**AIR) preparation derived from separately grown plant material.

### 2.2 Isolation of homozygous T-DNA insertion lines

Genomic DNA was extracted from *Arabidopsis* seedlings (Col-0 and mutant lines) using a Plant Genomic DNA Extraction Kit (Favorgen Biotech Corp., Taiwan). Homozygous mutant plants were identified by PCR using gene-specific primers designed via the SIGnAL T-DNA Primer Design tool (http://signal.salk.edu/tdnaprimers.2.html) in combination with a T-DNA left-border primer (Table 1 and Supplementary Table S1). PCR was performed under the following conditions: initial denaturation at 94 °C for 3 min; 35 cycles of 94 °C for 30 s, 55–60 °C for 45 s (annealing temperature adjusted according to primer Tm), and 72 °C for 1 min 15 s; and a final extension at 72 °C for 5 min.

### 2.3 Preparation of AIR and polysaccharide extraction

Cell wall material was prepared as AIR. Rosette leaves from 3-week-old plants and inflorescence stems from 5-week-old plants were harvested and ground to a fine powder in liquid nitrogen. The powder was suspended in 80% (*v*/v) ethanol and the residue was collected by centrifugation. The pellet was washed sequentially with 80% (*v*/v) ethanol (at least three times, or until the supernatant was colourless), absolute ethanol, acetone, and methanol, and then air-dried overnight. The AIR was further dried at 50–60 °C for 3 days. All genotypes within a mutant group were processed in parallel using identical extraction volumes, incubation times, and conditions. A pectin-enriched fraction was extracted by incubating AIR in 50 mM cyclohexane diamine tetraacetic acid (CDTA), pH 7.0, with shaking at room temperature overnight. The supernatant was filtered through nylon mesh and collected. The remaining residue was washed three times with distilled water and subsequently extracted with 4 M NaOH containing 1% (*w*/*v*) NaBH₄ at room temperature overnight to obtain a hemicellulose-enriched fraction. Both fractions were dialysed against distilled water at room temperature overnight and then lyophilised. Sequential extraction was used to obtain a CDTA-soluble pectin-enriched fraction followed by a NaOH-soluble hemicellulose-enriched fraction for subsequent GPC-ELISA profiling. The CDTA and NaOH extracts were weighed after lyophilisation, and extraction yields were recorded as mg extract and as percentage of AIR.

### 2.4 Gel permeation chromatography–enzyme-linked immunosorbent assay (GPC-ELISA)

Lyophilised fractions were dissolved in ultrapure water at 10 mg/mL and clarified by filtration through a 0.22 μm membrane. Samples were injected via a 500 μL sample loop and separated by gel permeation chromatography (GPC) on a Superose 6 Increase 10/300 GL column (Cytiva) using ultrapure water as the mobile phase at a flow rate of 0.5 mL/min; 0.5 mL fractions were collected. Dextran molecular weight standards (150 kDa and 410 kDa) and glucose (as a low-molecular-weight marker) were prepared at 1 mg/mL in ultrapure water and separated under identical conditions. Dextran-containing fractions were identified colourimetrically using the anthrone assay: 50 μL of each fraction was mixed with 150 μL of cold anthrone reagent (0.2% (w/v) anthrone in concentrated sulfuric acid) in a 96-well plate and incubated at 90 °C for 20 min. For immunochemical detection, GPC fractions were analysed by ELISA using the following primary antibodies: LM15 (XyG), LM19 (unesterified HG), LM20 (methyl-esterified HG), LM21 (HM), and LM28 (GX). Antibodies were originally obtained from PlantProbes (Leeds, UK) and are now available from Kerafast (Boston, MA, USA), Megazyme (Bray, Ireland), and Ximbio (London, UK). LM20 was used for CDTA fractions only, because alkaline NaOH extraction disrupts methyl esters and therefore precludes reliable detection of methyl-esterified HG in the NaOH fractions. Anti-rat IgG conjugated to horseradish peroxidase (HRP) was used as the secondary antibody. Polysaccharide standards, tamarind xyloglucan, polygalacturonic acid (PGA) from citrus pectin, apple pectin, carob galactomannan, and 4-*O*-methyl-D-glucurono-D-xylan, were dissolved in ultrapure water across a concentration range of 0.64 ng/mL to 10 μg/mL and included as internal standards for quantification against their respective primary antibodies using four-parameter logistic (4PL) standard curves. Each biological replicate was analysed by a separate GPC run, and ELISA was performed in technical duplicate for each fraction.

For ELISA, 50 μL of GPC fractions, polysaccharide standards, and a blank control (ultrapure water) were loaded onto flat-bottom 96-well plates (Costar 3599, Corning) and dried overnight at 37 °C. Wells were blocked with 200 μL of 3% (*w*/*v*) bovine serum albumin (BSA) in phosphate-buffered saline (PBS) at 37 °C for 1 h, after which the blocking solution was removed. Primary antibodies were diluted 1:50 in 1% (*w*/*v*) BSA/PBS, and 25 μL was added per well and incubated at 37 °C for 1 h. Plates were washed three times with 300 μL PBS using a microplate washer, then incubated with 50 μL of secondary antibody (1:5000 in 1% (*w*/*v*) BSA/PBS) at 37 °C for 1 h, followed by six washes with 300 μL PBS. Colour development was initiated by adding 50 μL/well of 3,3′,5,5′-tetramethylbenzidine (TMB) substrate solution and stopped with 50 μL/well of 0.5 N sulfuric acid after 15 min (LM15) or 20 min (LM19, LM20, LM21, and LM28). Absorbance was measured at 450 nm and 650 nm using a microplate reader. The relative amounts of each polysaccharide in GPC fractions were quantified against the corresponding 4PL standard curve. Technical duplicate ELISA readings were averaged to obtain one OD value per fraction for each biological replicate. Variation among biological replicates was calculated from the three independent biological-replicate values per genotype and fraction. Data were visualized as heat maps and chromatogram overlays using R version 4.4.1.

### 2.5 Statistical analysis

Statistical analyses were performed in R version 4.4.1. Technical duplicate ELISA readings were averaged to obtain one value for each GPC fraction and biological replicate. Two complementary *t*-test-based approaches were used to compare polysaccharide size-distribution profiles between each mutant and its concurrently grown, extracted, and analysed wild-type control. For the region-level analysis, GPC fractions were grouped into three predefined molecular-size regions based on the elution positions of the molecular-mass standards: high molecular size (fractions 8–19), intermediate molecular size (fractions 20–31), and low molecular size (fractions 32–43). For each antibody, extraction fraction, genotype, and biological replicate, the mean epitope signal across the fractions within each molecular-size region was calculated. Mutant and WT values were compared using two-tailed Welch's *t*-tests applied to the three biological-replicate bin means (*n* = 3 per genotype). For each mutant–WT comparison, the three bin-level *p*-values were adjusted within each antibody–extraction combination using the Benjamini–Hochberg false-discovery-rate procedure. Differences were considered statistically significant at an adjusted *p* < 0.05. For comparisons at individual fractions, two-tailed Student's *t*-tests were performed using the three biological replicates per genotype, with statistical significance set at *p* < 0.05. The region-level and individual-fraction analyses were used complementarily to identify differences across broader molecular-size regions and localised differences within the profiles, respectively. CDTA and NaOH extraction yields were compared between each mutant and its concurrently processed WT control using two-tailed Student's *t*-tests, with statistical significance set at *p* < 0.05.

To assess the contribution of different sources of variability to the WT reproducibility data, a variance components analysis was performed for each antibody–extraction–tissue combination using a one-way random-effects model with experiment as the random factor. For each antibody–extraction–tissue combination, variance components were estimated separately for each GPC fraction using a one-way random-effects model. The fraction-specific between-experiment and within-experiment (biological replicate) variance components were then pooled across fractions and expressed as the percentage contribution of each component to the total variance. The intraclass correlation coefficient (ICC) was calculated as the ratio of between-experiment variance to total variance.

## 3 Results

### 3.1 Reproducibility of GPC-ELISA profiling and extraction yields

All mutant–WT comparisons were performed within experimental groups in which the matched WT and mutant plants were grown, extracted, and analysed in parallel. Under our growth conditions, all 12 mutants were morphologically comparable to wild-type Col-0 at both the rosette (3-week) and mature bolting stages, showing no severe developmental defects or altered flowering time that would confound tissue sampling (Table 1 and Supplementary Fig. S1). Because WT controls were independently prepared in six experiments, comprising three inflorescence-stem and three rosette-leaf experiments, these datasets also allowed us to assess the reproducibility of GPC-ELISA profiling across independent experiments. For each antibody–extraction combination, we calculated pairwise Pearson correlations between individual biological-replicate WT profiles and examined coefficients of variation (Supplementary Fig. S2 and S3). The mean within-tissue correlation was *r* = 0.74, compared with an overall mean of *r* = 0.67 across both tissue types. Correlations among biological replicates within the same experiment were higher (mean *r* = 0.83), indicating good agreement among concurrently processed replicates. The lowest within-tissue correlations were observed for LM15 and LM21 in the CDTA fraction (*r* ≈ 0.46), where XyG and HM signals were close to the detection limit. By contrast, the well-resolved NaOH-fraction antibody–extraction combinations showed strong within-tissue agreement (LM15, LM19, and LM21; *r* ≈ 0.89–0.92), although LM28 in the NaOH fraction was comparatively more variable (within-tissue *r* = 0.73; overall *r* = 0.38), reflecting the tissue-dependent nature of GX extractability. Variation among biological replicates was lower for the signal-bearing NaOH-fraction epitopes (LM15, LM19, LM21, and LM28; CV approximately 18–30%) than for the CDTA fraction, where the CV for the reliably detected LM19 and LM20 epitopes was approximately 32–49%. A variance components analysis further quantified these sources of variability showing that, across all antibody–extraction–tissue combinations, variance was approximately equally partitioned between experiments (mean 46%) and within experiments (mean 54%), with the NaOH fraction showing a somewhat greater between-experiment contribution (mean 53%) than the CDTA fraction (mean 41%). Near-background signals, such as LM21 and LM28 in the CDTA fraction of leaf tissue, were dominated by within-experiment variance (>80%), consistent with measurement noise at low signal levels (Supplementary Table S2). Together, these results indicate that GPC-ELISA profiles were reproducible for epitopes that were sufficiently extracted and detected, whereas profiles associated with near-background signals were less reliable.

To assess whether differences in GPC-ELISA profiles could be influenced by variation in polymer extraction, the yields of the sequential CDTA and NaOH fractions were compared between each mutant and its concurrently processed WT control (Table 2 and Supplementary Table S3). Of the 12 mutants examined, eight showed no significant difference in either CDTA or NaOH extraction yield. CDTA yields were unchanged across all mutants, indicating broadly comparable recovery of the pectin-enriched fraction. By contrast, NaOH yields were significantly reduced in four mutants: *gux1-1* (55% of WT, *p* < 0.001), *gux2-2* (74% of WT, *p* < 0.01), *csla2-1* (90% of WT, *p* < 0.05), and *tbl2* (81% of WT, *p* < 0.05). Thus, extraction yields were broadly comparable between mutants and WT, although the reduced NaOH recovery in these four lines, particularly *gux1-1* and *gux2-2*, was considered when interpreting their GPC-ELISA profiles.

**Table 2:** Sequential extraction yields of CDTA- and NaOH-soluble cell wall fractions from 12 mutants and their matched wild-type controls. Values are means ± SD (*n* = 3) from three biological replicates and are expressed as percentages of alcohol-insoluble residue (AIR). Total yield represents the combined CDTA and NaOH yields. Group numbers identify genotypes that were grown, extracted, and analysed together with the same matched Col-0 control. Mutant and WT values were compared within each experimental group using two-tailed Student's *t*-tests. Asterisks indicate significant differences from the matched WT: *p < 0.05, **p < 0.01, and ***p < 0.001.

Group	Genotype	Tissue	CDTA (% AIR)	NaOH (% AIR)	Total (% AIR)
1	Col-0	stem	20.1 ± 1.8	24.4 ± 4.8	44.5 ± 6.3
1	*irx14L*	stem	23.9 ± 1.6	23.5 ± 1.9	47.4 ± 3.1
2	Col-0	stem	23.6 ± 0.4	20.2 ± 0.6	43.9 ± 0.2
2	*gux1-1*	stem	22.8 ± 3.6	11.0 ± 0.6***	33.9 ± 4.2
2	*gux2-2*	stem	21.5 ± 2.1	15.0 ± 1.8**	36.5 ± 3.6
3	Col-0	stem	26.2 ± 2.6	17.4 ± 7.7	43.6 ± 7.1
3	*irx15*	stem	24.9 ± 1.9	19.1 ± 0.4	44.0 ± 1.5
4	Col-0	leaf	23.0 ± 1.6	21.9 ± 0.5	44.9 ± 2.1
4	*csla2-1*	leaf	21.2 ± 1.4	19.8 ± 0.7*	41.1 ± 1.0
4	*tbl2*	leaf	21.3 ± 0.9	17.8 ± 1.9*	39.1 ± 2.3
4	*tbl3*	leaf	21.6 ± 1.6	16.9 ± 5.1	38.5 ± 6.7
5	Col-0	leaf	23.2 ± 0.7	19.7 ± 6.4	42.9 ± 6.6
5	*gaut10-2*	leaf	23.6 ± 0.8	29.8 ± 3.2	53.4 ± 3.3
5	*gaut11-2*	leaf	24.2 ± 0.8	22.6 ± 3.8	46.8 ± 2.9
5	*tbr*	leaf	23.0 ± 0.9	25.9 ± 0.5	48.8 ± 1.4
6	Col-0	leaf	23.3 ± 1.7	31.5 ± 1.3	54.8 ± 2.1
6	*axy4-4*	leaf	23.7 ± 1.4	29.1 ± 1.7	52.8 ± 1.2
6	*tbl31*	leaf	22.1 ± 2.6	28.0 ± 4.4	50.1 ± 3.1

### 3.2 GPC-ELISA profiling of xylan biosynthesis mutants

**Fig. 1:**
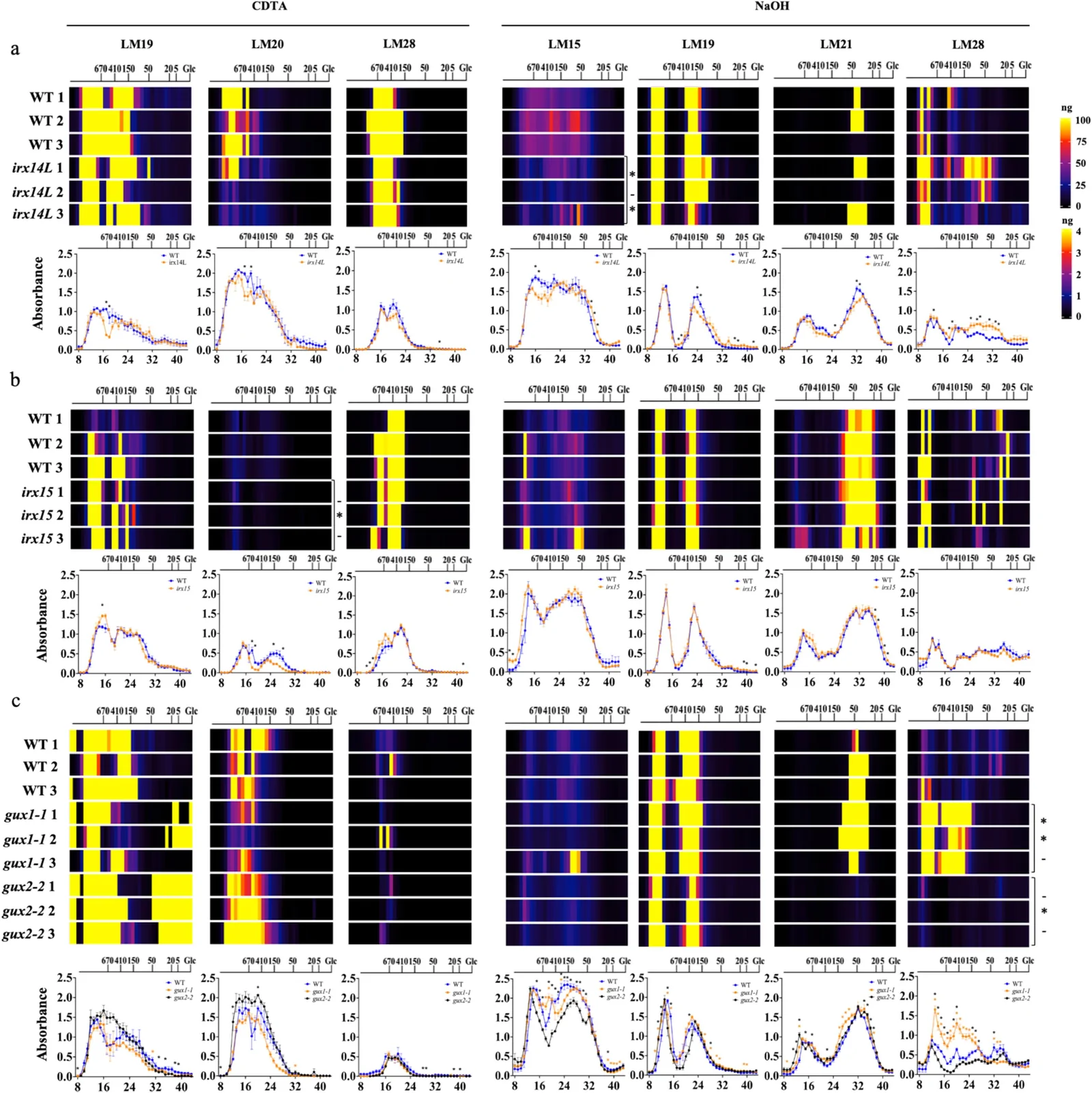
GPC-ELISA profiling of xylan biosynthesis mutants. GPC-ELISA profiles of sequential cell wall extracts from inflorescence stems of xylan-related mutants and their matched wild-type Col-0 controls: (a) *irx14L*, (b) *irx15*, and (c) *gux1-1* and *gux2-2*. Each panel represents an independently grown and processed mutant–WT experimental group. Cell wall material was sequentially extracted with CDTA (left) and NaOH (right). Fractions were analysed using LM15 for xyloglucan (XyG), LM19 for unesterified homogalacturonan (HG), LM20 for methyl-esterified HG, LM21 for heteromannan (HM), and LM28 for glucuronoxylan (GX). Within each panel, heat maps show the estimated quantity of each polysaccharide epitope per GPC fraction for three biological replicates (*n* = 3), each derived from an independent cell wall extraction. Colour scales range from 0 to 100 ng for all antibodies except LM21, for which the scale ranges from 0 to 4 ng. Chromatogram overlays show mean ± SE for the matched WT and mutant profiles. The *x*-axis indicates GPC fraction number, with molecular mass decreasing from left to right; approximate molecular-mass markers are shown above the profiles. For the region-level analysis, fractions were grouped into high- (fractions 8–19), intermediate- (fractions 20–31), and low-molecular-size regions (fractions 32–43). Mutant and WT biological-replicate bin means were compared using two-tailed Welch's *t*-tests, with the three bin-level *p*-values within each antibody–extraction combination adjusted using the Benjamini–Hochberg procedure. The three symbol rows represent, from top to bottom, the high-, intermediate-, and low-molecular-size bins, respectively; an asterisk indicates adjusted *p* < 0.05. Asterisks on the chromatogram overlays indicate individual fractions differing between mutant and matched WT by Student's *t*-test (*p* < 0.05).

To investigate how disruption of GX biosynthesis affects the molecular size distribution of other non-cellulosic wall polymers, we analysed inflorescence stem tissue, a secondary-wall, xylan-rich tissue, from four xylan-related mutants, *irx14L*, *irx15*, *gux1-1*, and *gux2-2*, using sequential CDTA and NaOH extraction followed by GPC-ELISA (*n* = 3 biological replicates per genotype, each from independent extractions). Fractions were probed with antibodies against XyG (LM15), HM (LM21), unesterified HG (LM19), methyl-esterified HG (LM20), and GX (LM28) (Fig. 1). Because alkaline extraction disrupts methyl esters, LM20 was omitted from the analysis of NaOH fractions. As expected for stem tissue, LM15 and LM21 signals were very low or absent in the CDTA fraction in WT and all mutants. These CDTA signals were therefore not informative for comparative interpretation. All mutant and WT comparisons were made within the same experiment using a matched WT control grown and extracted in parallel.

The *irx14L* mutant, which affects xylan backbone elongation, showed relatively minor changes in the CDTA fraction. LM19, LM20, and LM28 were broadly similar to WT, although those for LM19 and LM20 showed reduced signal at selected mid-size fractions (Student's *t*-test, *p* < 0.05), indicating that the pectic fraction was only modestly altered. In the NaOH fraction, LM15 was significantly reduced in the high- and low-molecular-size regions (adjusted *p* < 0.05). LM28 was elevated at selected individual fractions (Student's *t*-test, *p* < 0.05), although no significant difference was detected across the predefined molecular-size regions. Thus, *irx14L* showed reduced XyG at the region level together with a localised increase in GX signal in the NaOH fraction.

The *irx15* mutant showed comparatively modest effects. In the CDTA fraction, LM19 profiles were broadly similar to WT, although signal in the highest molecular-size range was slightly elevated. LM20 was reduced in the intermediate-molecular-size region, supported by both profile-level and fraction-wise comparisons (adjusted *p* < 0.05; Student's *t*-test, *p* < 0.05). LM28 showed a slight increase at the highest molecular-size fractions, but the overall GX profile was not clearly different from WT. In the NaOH fraction, LM15, LM19, LM21, and LM28 profiles were all broadly similar to WT, with no clear overall differences detected. Together, these data indicate that *irx15* has only a modest effect on matrix polysaccharide size distribution, with the clearest change being a reduction in methyl-esterified HG in the CDTA extract.

The two glucuronidation mutants, *gux1-1* and *gux2-2*, displayed the most contrasting GX profiles. In the CDTA fraction, neither mutant differed significantly from WT in any molecular-size region for LM19, LM20, or LM28, although the profiles showed local variation at selected individual fractions. *gux1-1* showed a reduction in LM19 across the large-to-mid molecular-size range, and LM20 also trended downward. By contrast, *gux2-2* showed the opposite tendency, in which LM19 and LM20 were increased, particularly in mid- and small-size fractions. Although both mutants disrupt xylan glucuronidation, they produced opposite HG responses in the CDTA fraction. In the NaOH fraction, *gux1-1* showed a marked increase in LM28 signal in the high- and intermediate-molecular-size regions (adjusted *p* < 0.05). By contrast, *gux2-2* showed a significant reduction in LM28 in the intermediate-molecular-size region (adjusted *p* < 0.05). In both mutants, LM15 was reduced relative to WT (Student's *t*-test, *p* < 0.05), although this did not reach statistical significance by binned *t*-test, and LM19 and LM21 showed only modest changes in distribution (Student's *t*-test, *p* < 0.05). These data indicate that loss of GUX1 and GUX2 function has divergent effects on the alkali-extractable wall matrix: *gux1-1* showed an increased GX signal within the NaOH fraction, whereas *gux2-2* showed a broad reduction in GX signal, together with reduced XyG and HG signal.

Taken together, the four xylan mutants demonstrate that perturbation of GX biosynthesis affects not only GX itself but also the size distribution and fraction-associated distribution of other wall polymers, particularly HG and XyG. The most pronounced and contrasting effects were associated with altered xylan glucuronidation, as *gux1-1* and *gux2-2* produced opposite changes in GX and distinct XyG and HG responses in the NaOH fraction. These results show that xylan backbone-associated and glucuronidation defects produce distinct cross-polymer profiles, depending on the particular biosynthetic or substitution step disrupted.

### 3.3 GPC-ELISA profiling of the *csla2-1* mannan biosynthesis mutant

**Fig. 2:**
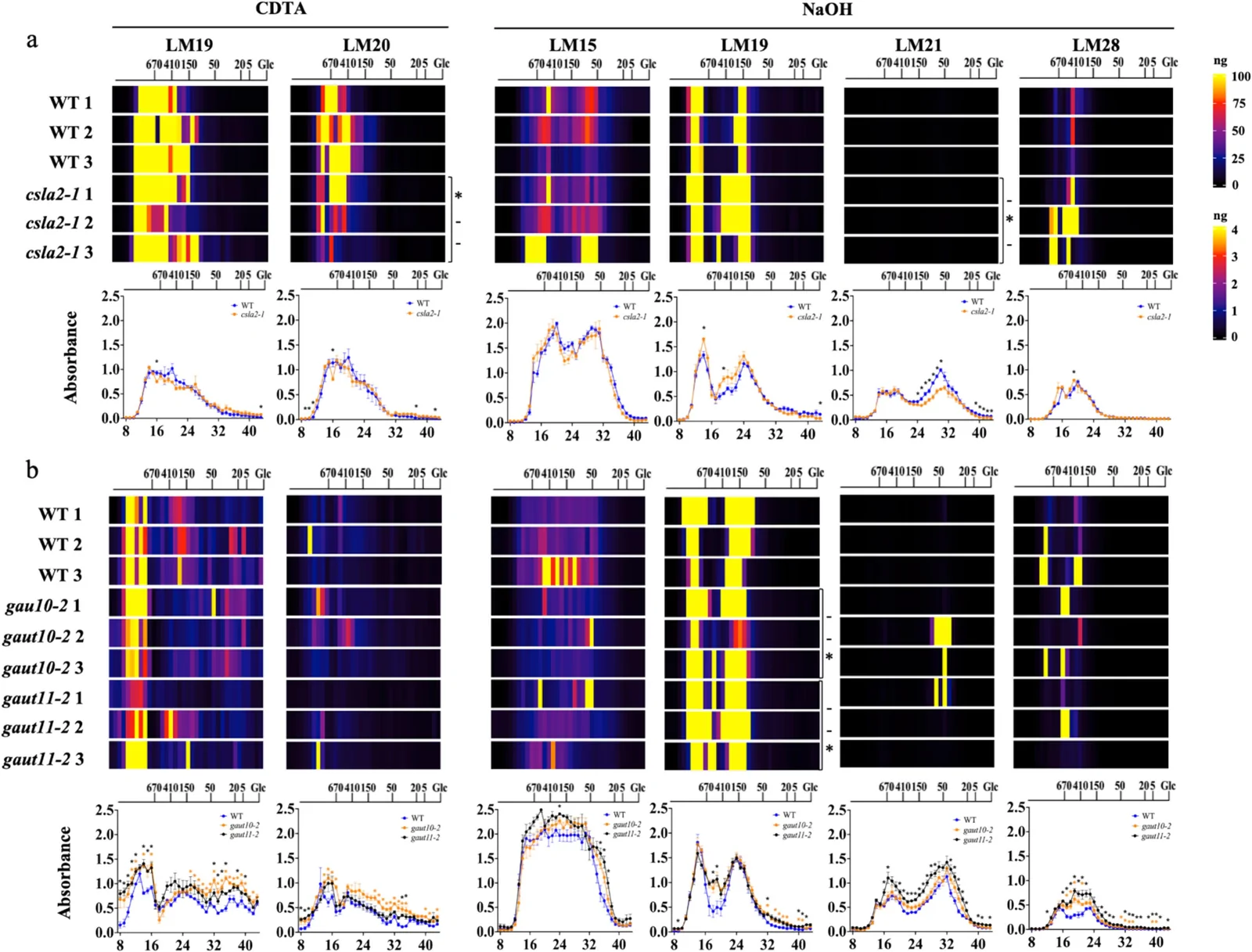
GPC-ELISA profiling of mannan and pectin biosynthesis mutants. GPC-ELISA profiles of sequential cell wall extracts from rosette leaves of the mannan biosynthesis mutant *csla2-1* and the pectin biosynthesis mutants *gaut10-2* and *gaut11-2*: (a) *csla2-1* and its matched wild-type Col-0 control and (b) *gaut10-2* and *gaut11-2* with their matched Col-0 control. Each panel represents an independently grown and processed mutant–WT experimental group. Within each panel, heat maps show epitope quantities across GPC fractions for three biological replicates per genotype (*n* = 3), and chromatogram overlays show mean ± SE. CDTA-derived pectin-enriched fractions are shown on the left and NaOH-derived hemicellulose-enriched fractions on the right. Antibody specificities, molecular-mass orientation, colour scales, molecular-size bins and statistical annotations are as described for Fig. 1.

To assess the effect of reduced HM on cell wall polysaccharide size distributions, we profiled rosette leaf tissue from the *csla2-1* mutant (*n* = 3 biological replicates). In the CDTA fraction, the LM19 and LM20 profiles in *csla2-1* were broadly similar to those of WT, though with a significant reduction in LM20 signal in the high-molecular-size region (adjusted *p* < 0.05) (Fig. 2). In the NaOH fraction, LM21 was reduced in *csla2-1*, particularly in the intermediate-molecular-size region (adjusted *p* < 0.05; Student's *t*-test, *p* < 0.05), consistent with the expected direct biosynthetic defect in HM. By contrast, LM15 and LM28 each showed broadly similar profiles between WT and *csla2-1*. LM19 displayed a modest increase in the large- to mid-size region (Student's *t*-test, *p* < 0.05). Thus, *csla2-1* showed the expected reduction in intermediate-molecular-size HM together with a secondary reduction in high-molecular-size methyl-esterified HG.

### 3.4 *gaut10-2* and *gaut11-2* pectin mutants show coordinated changes in HG and hemicellulose profiles

To investigate the effect of altered HG biosynthesis on cell wall polymer organisation, we analysed 3-week-old leaf tissue from the T-DNA insertion mutants *gaut10-2* and *gaut11-2*, which disrupt GAUT10 and GAUT11 galacturonosyltransferases involved in HG biosynthesis (*n* = 3 biological replicates). In the CDTA extract, both *gaut10-2* and *gaut11-2* showed elevated LM19 and LM20 signals relative to WT across several individual fractions (Student's *t*-test, *p* < 0.05), although no significant differences were detected in the three molecular-size regions (Fig. 2). These fraction-level results indicate similar local increases in both unesterified and methyl-esterified HG. In the NaOH extract, both mutants showed a significant increase in low-molecular-size unesterified HG detected by LM19 (adjusted *p* < 0.05), together with differences at selected individual fractions (Student's *t*-test, *p* < 0.05). LM15, LM21, and LM28 signals were also elevated at selected fractions across their profiles (Student's *t*-tests, *p* < 0.05). Overall, the two HG mutants showed increased low-molecular-size unesterified HG together with localised changes in XyG, HM, and GX profiles.

### 3.5 GPC-ELISA profiling of esterification-related mutants

**Fig. 3:**
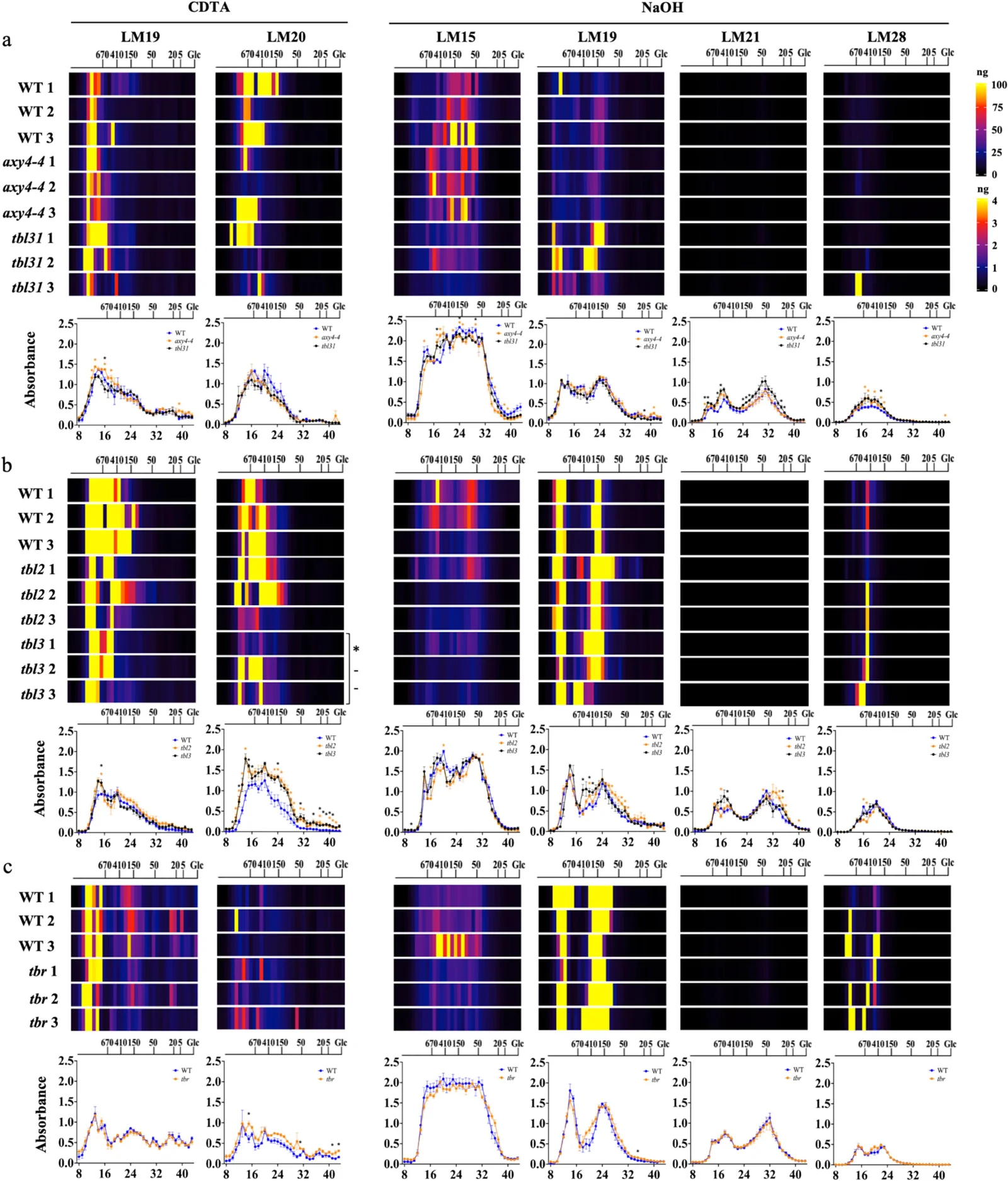
GPC-ELISA profiling of esterification-related mutants. GPC-ELISA profiles of sequential cell wall extracts from rosette leaves of five esterification-related mutants and their matched wild-type Col-0 controls: (a) *axy4-4* and *tbl31*, (b) *tbl2* and *tbl3*, and (c) *tbr*. Each panel represents an independently grown and processed mutant–WT experimental group; mutants presented within the same panel were compared with the same concurrently processed WT control. Within each panel, heat maps show epitope quantities across GPC fractions for three biological replicates per genotype (*n* = 3), and chromatogram overlays show mean ± SE. CDTA-derived pectin-enriched fractions are shown on the left and NaOH-derived hemicellulose-enriched fractions on the right. Antibody specificities, molecular-mass orientation, colour scales, molecular-size bins and statistical annotations are as described for Fig. 1.

We analysed five mutants affecting wall esterification or related wall assembly, including *axy4-4*, defective in XyG *O*-acetylation; *tbl31*, associated with xylan *O*-acetylation; *tbl2* and *tbl3*, members of the DUF231/TBL family implicated in wall acetylation and wall assembly; and *tbr*, a TBL-family mutant linked to trichome cellulose deposition and pectic esterification (Bischoff et al., 2010; Gille et al., 2011; Xiong, Cheng, & Pauly, 2013; Yuan et al., 2016). Rosette leaves from 3-week-old plants were used for GPC-ELISA profiling (*n* = 3 biological replicates) (Fig. 3). *axy4-4* and *tbl31* showed the most similar overall profiles. In the CDTA fraction, LM19 and LM20 were broadly comparable to WT, although several individual fractions differed from WT by Student's *t*-test (*p* < 0.05). Thus, disruption of XyG *O*-acetylation in *axy4-4* or xylan *O*-acetylation in *tbl31* had only modest effects on unesterified HG and methyl-esterified HG signals in the CDTA fraction. In the NaOH fraction, LM15 and LM19 profiles in *axy4-4* and *tbl31* were also broadly similar to WT, although selected mid-to-low fractions differed (Student's *t*-test, *p* < 0.05). By contrast, both LM21 and LM28 were slightly elevated in *axy4-4* and *tbl31* at selected fractions (Student's *t*-test, *p* < 0.05). In both *axy4-4* and *tbl31*, the clearest deviations from WT were increased HM and GX signals in the NaOH fraction, whereas HG remained comparatively similar to WT.

*tbl2* and *tbl3* also showed closely related profiles, but these differed from those of *axy4-4* and *tbl31*. In the CDTA fraction, LM19 was elevated at the large-size peak in both mutants (Student's *t*-test, *p* < 0.05). LM20 showed overall increases, with significant differences at selected fractions in both *tbl2* and *tbl3* (Student's *t*-test, *p* < 0.05). In the region-level analysis, only *tbl3* showed a significant increase in LM20 signal in the high-molecular-size region (adjusted *p* < 0.05). In the NaOH fraction, LM15 was broadly similar to WT in both mutants. LM19 increased in the mid-to-small size range at selected fractions (Student's *t*-test, *p* < 0.05). LM21 differed between the two mutants: *tbl2* showed reduced signal at the large-size range and increased signal at the small-size range, whereas *tbl3* showed increased signal at large sizes (Student's *t*-test, *p* < 0.05). The *tbl3* mutant also showed some reduction in LM28 in large fractions. Overall, *tbl2* and *tbl3* showed broad increases in methyl-esterified HG-associated signal in the CDTA fraction. These increases were detected at multiple individual fractions in both mutants, while *tbl3* additionally showed a significant increase in the high-molecular-size region. The *tbr* mutant showed a more restricted phenotype than the other mutants in this group. In the CDTA fraction, LM19 profiles were essentially unchanged relative to WT across all fractions. LM20 was elevated in *tbr* at selected fractions (Student's *t*-test, *p* < 0.05), indicating a modest increase in methyl-esterified HG in the CDTA fraction. In the NaOH fraction, LM15, LM19, LM21, and LM28 profiles were all broadly similar to WT, with no strong overall differences detected. Thus, *tbr* showed a moderate, localised increase in methyl-esterified HG at selected CDTA fractions, while the NaOH profiles were largely unaffected.

Cross-mutant comparison resolved three response classes. First, *axy4-4* and *tbl31* were defined by the clearest deviations of HM and GX in the NaOH fraction. Second, *tbl2* and *tbl3* were defined by strong increases in methyl-esterified HG in the CDTA fraction, with limited and variable changes in unesterified HG, HM, and GX in the NaOH fraction. Third, *tbr* showed the weakest overall phenotype, limited mainly to a modest increase in methyl-esterified HG in the CDTA fraction. Across the mutant set, alterations in HG, particularly methyl-esterified HG, were the most frequent response, whereas changes in HM and GX were more pathway-specific.

## 4 Discussion

Although cell wall mutants are often interpreted primarily in terms of the polymer directly affected, the wall itself is an integrated matrix in which changes in one component may influence the organisation of others (Alonso Baez & Bacete, 2023; Cosgrove, 2023). The present data support this broader view by showing that alteration of a single polysaccharide biosynthesis or modification pathway can affect the molecular-size distributions of non-target matrix polysaccharides. In our mutant panel, these secondary effects were most evident for xylan- and pectin-related pathways, whereas the mannan mutant showed comparatively limited changes. In several cases, GPC-ELISA resolved these changes more clearly than bulk compositional analyses by capturing differences across sequentially extracted wall fractions and molecular-size classes. Because such changes are likely to influence wall mechanics, cell expansion, and biomass properties, they are relevant not only to matrix organisation itself but also to plant growth and cell wall utilisation. It should be noted that GPC-ELISA was applied to sequentially extracted fractions rather than unfractionated wall material, so differences in epitope signal may reflect changes in polymer abundance, partitioning, molecular size distribution or a combination of these effects within a given extractable wall fraction rather than total wall abundance alone. In addition, the study relies primarily on a single profiling method. Complementary analyses, such as monosaccharide composition analysis, glycome profiling, solid-state NMR, or mechanical testing, would be required to determine whether the observed redistributions reflect structural changes in wall architecture. Further assessments of cell wall integrity signalling, mechanical properties, or the spatial distribution of epitopes within the wall would help distinguish among alternative mechanistic explanations for the observed cross-polymer effects.

Because mutations that alter wall composition may also alter extractability, differences in epitope signal could reflect changes in polymer extractability rather than absolute abundance. The use of sequential extraction with defined conditions, with all genotypes within a group processed in parallel, helps distinguish true biological redistribution from extraction artifacts. The reduced NaOH recovery in *gux1-1* and *gux2-2* is consistent with their defects in xylan glucuronidation, because xylan substitution can influence polymer solubility and extractability under alkaline conditions (Bromley et al., 2013; Mortimer et al., 2010). Their LM28 profiles may therefore reflect effects of the respective glucuronidation defects on both GX extractability and molecular-size distribution. Importantly, however, the two mutants showed contrasting GX profiles despite both having reduced NaOH yields: *gux1-1* displayed a marked increase in LM28 signal within the NaOH-soluble fraction, whereas *gux2-2* showed broad depletion. A general reduction in extraction efficiency would be expected to affect both mutants in the same direction, suggesting that the opposing profiles cannot be explained solely by reduced extraction yield. Nevertheless, their LM28 profiles should be interpreted as changes within the extractable GX pool rather than as direct measures of total wall GX abundance. The smaller reductions in *csla2-1* and *tbl2* are likewise consistent with perturbation of their directly affected biosynthetic or modification pathways. More broadly, the mutants examined here lacked severe developmental or growth phenotypes, and extraction yields were comparable with WT for most lines. This supports interpretation of the non-target epitope changes as being consistent with specific cross-polymer effects, while recognising that complementary analytical methods would be required to exclude extractability-related explanations fully.

The backbone-associated mutant *irx14L* produced relatively modest changes in the CDTA fraction but clearer changes in the NaOH fraction. The targeted xylan phenotype was an elevation of GX-associated LM28 signal at selected individual fractions in the NaOH extract, consistent with a partial defect in xylan backbone elongation, in line with the known partial redundancy between IRX14 and IRX14L in GX synthesis (Keppler & Showalter, 2010; Lee, Teng, Zhong, & Ye, 2012b; Mortimer et al., 2015; Wu et al., 2010). Secondary effects on non-target polymers included a reduction in XyG in the high- and low-molecular-size regions of the NaOH fraction and modest redistribution of unesterified HG in the CDTA fraction. The *irx15* mutant showed a milder phenotype overall, with the clearest change being a reduction in methyl-esterified HG in the CDTA fraction and little evidence of strong shifts in the NaOH fraction. This is consistent with IRX15 acting as an accessory factor in xylan deposition rather than as a catalytic subunit of the xylan synthase machinery (Brown et al., 2011; Jensen et al., 2011). These accompanying changes in non-xylan polymers suggest that even a relatively mild xylan backbone defect can influence the molecular-size distribution of other non-target matrix polysaccharides, but generally more modestly than the glucuronidation mutants.

The strongest and most informative xylan phenotypes were observed in the glucuronidation mutants *gux1-1* and *gux2-2*. The targeted polymer effects were most clearly seen in the NaOH fraction: *gux1-1* displayed a marked increase in GX signal, whereas *gux2-2* showed broad depletion of GX signal. These opposite effects on LM28 signal within the NaOH fraction are particularly informative because they support the proposed distinct roles of GUX1 and GUX2 in xylan glucuronidation. GUX1 catalyses evenly spaced GlcA substitution, which permits xylan to adopt the two-fold screw conformation that is compatible with hydrogen bonding to cellulose microfibril surfaces (Busse-Wicher et al., 2014; Grantham et al., 2017). GUX2, by contrast, catalyses more irregularly spaced GlcA substitution, which disrupts this regular conformation and reduces xylan–cellulose binding (Simmons et al., 2016; Wierzbicki, Maloney, Mizrachi, & Myburg, 2019). In *gux1-1*, loss of the predominantly even-spaced GUX1-dependent glucuronidation pattern leaves xylan enriched in GUX2-dependent, more irregularly substituted domains. This altered substitution pattern may weaken or modify xylan–cellulose association and is consistent with the increased representation of GX-associated LM28 signal within the NaOH fraction. Conversely, in *gux2-2*, retention of the predominantly GUX1-dependent, regularly substituted xylan domain may favour cellulose association, consistent with the reduced LM28 signal in the NaOH fraction. Importantly, both mutants showed reduced NaOH extraction yields (*gux1-1*: 55% of WT, *p* < 0.001 and *gux2-2*: 74%, *p* < 0.01), yet their LM28 profiles changed in opposite directions, indicating that the contrasting profiles cannot be explained by a general reduction in extraction yields alone. These results also support the validity of the GPC-ELISA method, as the technique is sensitive enough to resolve GX profile differences that are consistent with the established GUX1/GUX2 functional model (Bromley et al., 2013; Lee, Teng, Zhong, & Ye, 2012a; Mortimer et al., 2010). Secondary effects on non-target polymers were also observed in the CDTA fraction, where *gux1-1* showed reduced unesterified HG and a downward trend in methyl-esterified HG, whereas *gux2-2* showed increased unesterified HG and a modest upward trend in methyl-esterified HG. In the NaOH fraction, *gux2-2* additionally showed reduced XyG and unesterified HG. Taken together, the glucuronidation mutants show that changes in xylan substitution produce stronger and more divergent cross-polymer effects than the milder backbone-associated defects, and that the direction of these effects is consistent with the proposed structural roles of GUX1 and GUX2.

By contrast, *csla2-1* showed a comparatively restricted phenotype. The expected direct effect was a reduced HM-associated signal in the NaOH fraction, especially in mid-to-small size fractions, consistent with the contribution of CSLA2 to Arabidopsis glucomannan biosynthesis (Goubet et al., 2009). Secondary changes in other polymers were modest, with little evidence for broad shifts in XyG or GX and only a limited change in HG. This restrained phenotype is consistent with partial redundancy among CSLA family members and with overlap between glucomannan and XyG function in primary walls (Yu et al., 2022). The *csla2-1* mutant therefore provides a useful contrast with the broader cross-polymer effects observed in xylan- and pectin-related mutants.

The *gaut10-2* and *gaut11-2* mutants provided some of the clearest evidence that perturbation of one matrix polymer can reshape several others. GAUT10 and GAUT11 are members of the GT8 galacturonosyltransferase family responsible for HG biosynthesis, and several GAUT proteins have been shown biochemically to synthesize polymeric HG through different mechanisms (Amos et al., 2018; Engle et al., 2021). This suggests that different GAUTs may generate distinct HG subpopulations, consistent with earlier GPC-ELISA profiling of related *gaut* mutants, including *gaut7-1*, *gaut8-1* and *gaut14-1*, which showed distinct HG redistribution signatures (Sathitnaitham et al., 2021). In our data, both *gaut10-2* and *gaut11-2* showed increased HG signals across much of the CDTA fraction rather than a simple reduction in pectic epitopes. Because GAUT10 and GAUT11 are biosynthetic enzymes, this pattern is more likely to reflect altered partitioning and/or fraction-associated recovery of HG than a straightforward increase in HG synthesis. Both mutants also displayed increased or broadened XyG, HM, and GX signals in the NaOH fraction, suggesting that perturbation of HG biosynthesis does not remain confined to the pectic network but is accompanied by broad changes in hemicellulose organisation. This interpretation is reinforced by Dash et al. (2023), who showed that *gaut10-3* roots display altered pectic epitopes together with altered hemicelluloses. More broadly, these phenotypes support the view that HG contributes to the organisation of other wall polymers, with consequences for the distribution of hemicelluloses between wall fractions and molecular-size classes.

This interpretation is consistent with broader evidence that pectin integrity influences the cellulose-hemicellulose scaffold. For example, the *qua2* pectin methyltransferase mutant shows impaired cellulose biosynthesis and altered wall integrity signalling (Du et al., 2020), and pectin side-chain architecture can participate directly in cellulose-associated wall interactions (Gao et al., 2023). Recent work on XyG-deficient seedlings has likewise shown that altered pectin accumulation can compensate for the loss of XyG-mediated mechanical properties (Daher et al., 2024). The broader relevance of pectin–xylan interdependence is also highlighted by studies in Populus, where increased xylan and homogalacturonan production through GAUT12 overexpression caused increased recalcitrance and decreased growth (Biswal, Atmodjo, Pattathil, et al., 2018), and where coordinated pectin and xylan biosynthesis has implications for biofuel production (Schultz & Coleman, 2021). Overall, these observations support a role for HG as an important organiser of matrix architecture, with consequences for hemicellulose partitioning and size distribution.

The mutants affecting XyG or xylan acetylation, or cell wall esterification-related pathways, did not produce a single shared response. Instead, they separated into distinct redistribution signatures, suggesting that wall esterification influences matrix organisation in pathway-specific ways. *axy4-4* and *tbl31* were characterised mainly by elevated HM and GX signals in the NaOH fraction, whereas HG in the CDTA fraction changed only modestly. This is consistent with altered interactions among acetylated hemicelluloses and cellulose-associated wall domains (Busse-Wicher et al., 2014; Keppler & Showalter, 2010; Simmons et al., 2016; Yuan et al., 2016; Zhong, Cui, & Ye, 2017; Zhu et al., 2014). *tbl2* and *tbl3*, by contrast, showed increases in methyl-esterified HG in the CDTA extract, with weaker and more variable effects on the NaOH fraction.

The *tbr* mutant showed the weakest overall response, with only a modest increase in methyl-esterified HG and little evidence for broader redistribution. Interpretation of these methyl-esterification-related changes is nevertheless limited by the absence of a pectin methylesterase mutant as a positive control. Inclusion of a well-characterised PME or PME-related mutants in future studies would help determine whether the observed shifts reflect genuine changes in pectin methyl-esterification, altered epitope accessibility, or redistribution of HG among extractable wall fractions. These distinctions do not support a single generic acetylation-defect phenotype and instead suggest that different members of the TBL family influence different parts of the wall network. In addition to hemicellulose acetylation, multiple TBL/POAT proteins have been shown to acetylate HG and RG-I (Stranne et al., 2018; Zhong et al., 2024; Zhong, Cui, Richardson, & Ye, 2023), so the phenotypes of *tbl2*, *tbl3*, and *tbr* may reflect altered esterification states of more than one matrix polymer.

**Fig. 4:**
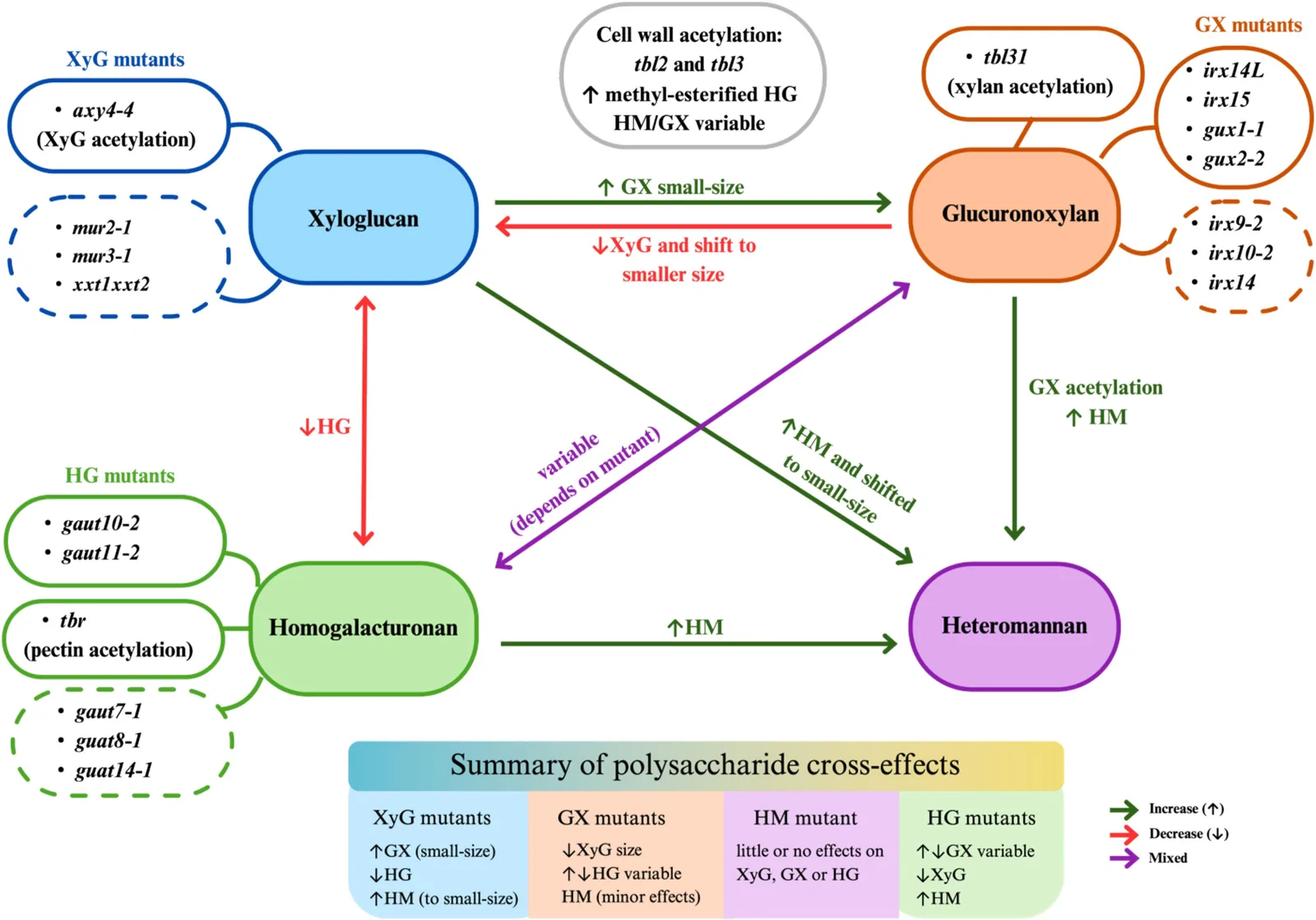
Schematic representation of the matrix polysaccharide coupling network inferred from GPC-ELISA profiles of all mutant groups. The four major matrix polysaccharides, xyloglucan, glucuronoxylan, homogalacturonan, and heteromannan, are shown together with the mutant classes affecting each pathway. Solid-outline boxes indicate mutants analysed in this study, whereas dashed-outline boxes indicate relationships supported by previously published mutant analyses. Arrows indicate secondary cross-polymer effects detected relative to wild type, including increases, decreases, or variable responses depending on the mutant examined. The network illustrates that perturbation of a single biosynthetic or modification pathway is associated with directional effects on other non-target polysaccharides within extractable wall fractions.

The GPC-ELISA profiles are consistent with a pattern in which perturbation of one polysaccharide pathway affects non-target matrix polysaccharides and their molecular-size distributions within extractable wall fractions. This coupling is not identical across all pathways, but instead supports a directional model of cross-polymer interdependence, as summarised in Fig. 4. A recurrent feature of the HG mutants was altered GX, XyG and HM signals in the NaOH fraction. By contrast, xylan-related mutants also influenced HG, but the outcome depended on the specific biosynthetic step affected. Backbone-related defects tended to reduce methyl-esterified HG or cause only modest redistribution, whereas the two *gux* mutants showed divergent HG responses. A further directional relationship was observed in *axy4-4* and *tbl31*, where altered XyG or xylan acetylation was associated with increased HM and GX signals in the NaOH fraction. Conversely, *tbl2* and *tbl3* showed increased HG-associated signal, whereas *csla2-1* showed only modest secondary changes. This network perspective is supported not only by the present mutant set, but also by earlier analyses of XyG-deficient mutants. *mur2*, *mur3*, and *xxt1 xxt2* all showed compensatory increases in HM and HG (Sathitnaitham et al., 2021; Sowinski et al., 2022), extending the coupling model to XyG as an additional node. Together, these comparisons reinforce the idea that different biosynthetic nodes differ in how strongly and in what direction they influence the broader matrix.

A key contribution of the present study is the direct comparison of mutants spanning four distinct matrix polysaccharide pathways: xylan biosynthesis and glucuronidation, mannan biosynthesis, pectin HG biosynthesis, and matrix polysaccharide *O*-acetylation. Earlier GPC-ELISA profiling studies have examined individual pathways or smaller mutant sets (Sathitnaitham et al., 2021), but a cross-pathway comparison of this breadth has not previously been reported. Although HG has long been recognised as an important organiser of cell wall architecture, the present findings extend this understanding by showing that disruption of HG biosynthesis is associated not simply with changes in pectin abundance, but with coordinated redistribution of GX, XyG, and HM across extractable wall fractions and molecular-size classes. The comparative analysis further indicates that these cross-polymer relationships are directional and asymmetric rather than reflecting a uniform compensatory response: perturbation of HG or GX pathways produced broad effects on other matrix polymers, whereas disruption of mannan biosynthesis caused comparatively limited secondary changes. The contrasting HG responses of *gux1-1* and *gux2-2* also suggest that the effect of xylan on pectin organisation depends on the specific glucuronidation pattern and xylan subpopulation affected, rather than simply on an overall reduction in glucuronic acid substitution. Similarly, the distinct redistribution profiles of the esterification-related mutants indicate that altered esterification does not generate a single generic response but influences matrix organisation in an enzyme- and polymer-specific manner. Together, these findings support a more differentiated model of cell wall interdependence in which HG and GX act as relatively influential nodes, while the direction and magnitude of secondary effects depend on the particular biosynthetic or modification step perturbed.

Overall, the comparative patterns support a model in which matrix-polysaccharide interactions are coordinated at multiple levels. GX and HG appear to be particularly influential components because perturbation of either pathway was associated with broad secondary effects on other polymers, whereas HM appeared less influential in the rosette leaf context examined here. Esterification-related pathways, meanwhile, produced distinct redistribution signatures depending on the affected enzyme and its target polysaccharide. These patterns may arise through both shared biosynthetic processes in the Golgi apparatus and post-secretory interactions within the apoplast (Driouich et al., 2012; Du et al., 2022; Grantham et al., 2017; Parsons et al., 2012; Simmons et al., 2016; Temple et al., 2022; Temple, Saez-Aguayo, Reyes, & Bhave, 2016). Perturbation of one pathway could alter biosynthetic balance or trafficking within the Golgi, while post-secretory effects may influence cellulose-associated space, wall hydration, polymer mobility, and cell wall integrity signalling. Xylan is required for proper cellulose microfibril bundling and alignment (Crowe et al., 2021), whereas pectin modification can influence cellulose biosynthesis and wall mechanics (Du et al., 2020; Gao et al., 2023). These interactions have practical implications for cell wall engineering and biomass utilisation because modifying one biosynthetic or modification pathway to alter recalcitrance, mechanics, digestibility, or stress resilience may produce broader effects than those predicted from the direct target alone. For example, reducing xylan content or altering xylan substitution may secondarily affect pectin organisation, whereas perturbing HG biosynthesis may reshape the partitioning and molecular-size distribution of several hemicelluloses.

## CRediT authorship contribution statement

**Supanut Utthiya:** Writing – original draft, Visualization, Investigation, Formal analysis, Data curation, Conceptualization. **Sukhita Sathitnaitham:** Investigation, Formal analysis. **Anongpat Suttangkakul:** Investigation, Formal analysis. **Passorn Wonnapinij:** Investigation, Formal analysis. **Seth J. Davis:** Writing – review & editing, Investigation. **Leonardo D. Gomez:** Writing – review & editing, Investigation. **Supachai Vuttipongchaikij:** Writing – review & editing, Writing – original draft, Visualization, Investigation, Conceptualization.

## Declaration of generative AI and AI-assisted technologies in the writing process

During the preparation of this work the author(s) used ChatGPT for language editing and proofreading. After using this tool/service, the author(s) reviewed and edited the content as needed and take(s) full responsibility for the content of the publication.

## Declaration of competing interest

The authors declare that they have no known competing financial interests or personal relationships that could have appeared to influence the work reported in this paper.

## Data Availability

All data generated and used in this study are available upon request or as Supporting Information for this article.
