# Supplementary material for: Mass distribution profiling of cell wall matrix polysaccharide biosynthesis mutants reveals alterations in non-targeted matrix polysaccharides
Source: Cell Surf. 2026 Sep 5;16:100181. doi: 10.1016/j.tcsw.2026.100181 (PMC13579315; doi:10.1016/j.tcsw.2026.100181)
Supplement: Supplementary file 1 [file mmc1.docx]

Supplementary Table S1. Mutant data and primers for screening homozygous mutants.

| Mutant | Forward primer (5’-3’) | Reverse primer (5’-3’) |
| --- | --- | --- |
| *irx14L* | CTTGCTCTTCGACACTCTTGG | ATCGATGTACGGTGTGAGGAG |
| *irx15* | TCAAACCAAGTTGTTTCCAGC | GAGAAGATTTTGCACAGGACG |
| *gux1-1* | CATAGCCGGAACTTGCTGTAG | TCTTTCCCACTCATCTTCTCG |
| *gux2-2* | GGAGGACTCCTAAGTCCGATG | TCTCCACGATGACTCCATCTC |
| *csla2-1* | TCAAGCATTGCGAGTATGTTG | ACACTACCAAGTGTCGGCAAG |
| *gaut10-2* | ATGGAACGAGCTCAAAACAAG | GCAGCACTCGATAAAAGCAAC |
| *gaut11-2* | CAATATTTGGGCAATTGGTTG | AATAGCCAGCTTTAGCCAAGG |
| *axy4-4* | CGAGCAACAAAATGTTCCAAG | TCACGTCCAACACCTCTAACC |
| *tbl2* | TCAACGAATAATGGGTCAAGC | GAGCCATTCTCTTTGCTTGTG |
| *tbl3* | TCATGTGCTTTATCACGCTTG | GAAGTGCTTGACGACAGGTTC |
| *tbl31* | CAGAGGTTTTAAACACTGCGC | TCTAGTAGTGGCTCAGGCTCG |
| *tbr* | ACGATTTTGCGTTTGAAAATG | ATCTGGGTTGGTTCCGTTTAC |
| SAIL LB | TTCATAACCAATCTCGATACAC |  |
| SALK LB | GCGTGGACCGCTTGCTGCAACTC |  |
| GABI LB | CCCATTTGGACGTGAATGTAGACA |  |

**Supplementary Table S2.** Variance components and individual-replicate correlation statistics for wild-type GPC-ELISA reproducibility analysis. Intraclass correlation coefficient: ICC.

| Extraction | Antibody | Tissue | Number of experiments | Variance  between experiments | Variance  within experiments | Total variance | % between exp | % within exp | ICC |
| --- | --- | --- | --- | --- | --- | --- | --- | --- | --- |
| CDTA | LM15 | stem | 3 | 0.069359 | 0.037863 | 0.107222 | 64.7 | 35.3 | 0.647 |
| CDTA | LM15 | leaf | 3 | 0.014489 | 0.014641 | 0.029131 | 49.7 | 50.3 | 0.497 |
| CDTA | LM19 | stem | 3 | 0.404101 | 1.560469 | 1.96457 | 20.6 | 79.4 | 0.206 |
| CDTA | LM19 | leaf | 3 | 1.300391 | 0.792166 | 2.092557 | 62.1 | 37.9 | 0.621 |
| CDTA | LM20 | stem | 3 | 7.667582 | 1.839469 | 9.507051 | 80.7 | 19.3 | 0.807 |
| CDTA | LM20 | leaf | 3 | 0.896232 | 1.03948 | 1.935712 | 46.3 | 53.7 | 0.463 |
| CDTA | LM21 | stem | 3 | 0.001875 | 0.050567 | 0.052442 | 3.6 | 96.4 | 0.036 |
| CDTA | LM21 | leaf | 3 | 0.002297 | 0.010872 | 0.013169 | 17.4 | 82.6 | 0.174 |
| CDTA | LM28 | stem | 3 | 0.875985 | 0.494139 | 1.370124 | 63.9 | 36.1 | 0.639 |
| CDTA | LM28 | leaf | 3 | 0.000789 | 0.035977 | 0.036766 | 2.1 | 97.9 | 0.021 |
| NaOH | LM15 | stem | 3 | 1.125463 | 2.01102 | 3.136483 | 35.9 | 64.1 | 0.359 |
| NaOH | LM15 | leaf | 3 | 2.254042 | 1.411704 | 3.665747 | 61.5 | 38.5 | 0.615 |
| NaOH | LM19 | stem | 3 | 0.633738 | 0.723013 | 1.35675 | 46.7 | 53.3 | 0.467 |
| NaOH | LM19 | leaf | 3 | 0.721774 | 0.672847 | 1.394621 | 51.8 | 48.2 | 0.518 |
| NaOH | LM21 | stem | 3 | 0.575165 | 0.346885 | 0.92205 | 62.4 | 37.6 | 0.624 |
| NaOH | LM21 | leaf | 3 | 0.244058 | 0.278823 | 0.522881 | 46.7 | 53.3 | 0.467 |
| NaOH | LM28 | stem | 3 | 0.451982 | 0.491461 | 0.943443 | 47.9 | 52.1 | 0.479 |
| NaOH | LM28 | leaf | 3 | 0.182074 | 0.074594 | 0.256668 | 70.9 | 29.1 | 0.709 |

**Supplementary Table S3. Extraction yields of sequential CDTA- and NaOH-soluble cell wall fractions from 12 mutants and their matched wild-type controls.** Each row represents one biological replicate derived from an independent alcohol-insoluble residue (AIR) preparation of approximately 0.5 g. Extraction yields are reported as recovered dry mass (mg) and as a percentage of the input AIR. Group numbers identify genotypes that were grown, extracted, and analysed together with the same matched Col-0 control.

| Group | Genotype | Tissue | AIR (mg) | CDTA (mg) | NaOH (mg) | CDTA  (% AIR) | NaOH  (% AIR) |
| --- | --- | --- | --- | --- | --- | --- | --- |
| 1 | WT | stem | 500 | 110 | 149 | 22.0 | 29.8 |
| 1 | WT | stem | 498 | 99 | 104 | 19.9 | 20.9 |
| 1 | WT | stem | 498 | 92 | 112 | 18.5 | 22.5 |
| 1 | *irx14L* | stem | 499 | 111 | 116 | 22.2 | 23.2 |
| 1 | *irx14L* | stem | 500 | 120 | 109 | 24.0 | 21.8 |
| 1 | *irx14L* | stem | 500 | 127 | 128 | 25.4 | 25.6 |
| 2 | WT | stem | 500 | 116.7 | 103.5 | 23.3 | 20.7 |
| 2 | WT | stem | 508 | 122.5 | 99.5 | 24.1 | 19.6 |
| 2 | WT | stem | 505 | 118.2 | 103.1 | 23.4 | 20.4 |
| 2 | *gux1-1* | stem | 503 | 95.8 | 51.9 | 19.0 | 10.3 |
| 2 | *gux1-1* | stem | 500 | 130.5 | 57.6 | 26.1 | 11.5 |
| 2 | *gux1-1* | stem | 500 | 116.7 | 56.5 | 23.3 | 11.3 |
| 2 | *gux2-2* | stem | 505 | 100.9 | 76.4 | 20.0 | 15.1 |
| 2 | *gux2-2* | stem | 502 | 103.5 | 66 | 20.6 | 13.1 |
| 2 | *gux2-2* | stem | 499 | 118.8 | 83.3 | 23.8 | 16.7 |
| 3 | WT | stem | 498.7 | 116.2 | 113.4 | 23.3 | 22.7 |
| 3 | WT | stem | 500.8 | 135.3 | 42.7 | 27.0 | 8.5 |
| 3 | WT | stem | 504 | 143.1 | 104.8 | 28.4 | 20.8 |
| 3 | *irx15* | stem | 505.5 | 127.9 | 95.7 | 25.3 | 18.9 |
| 3 | *irx15* | stem | 500 | 132.4 | 94.2 | 26.5 | 18.8 |
| 3 | *irx15* | stem | 502 | 114.3 | 98.3 | 22.8 | 19.6 |
| 4 | WT | leaf | 500.1 | 109 | 107.9 | 21.8 | 21.6 |
| 4 | WT | leaf | 500.1 | 112.3 | 108.6 | 22.5 | 21.7 |
| 4 | WT | leaf | 502.8 | 124.9 | 112.9 | 24.8 | 22.5 |
| 4 | *csla2-1* | leaf | 504.5 | 108.8 | 102.2 | 21.6 | 20.3 |
| 4 | *csla2-1* | leaf | 501.5 | 99 | 101.1 | 19.7 | 20.2 |
| 4 | *csla2-1* | leaf | 511.6 | 114.5 | 97.5 | 22.4 | 19.1 |
| 4 | *tbl2* | leaf | 500.6 | 109.7 | 99.4 | 21.9 | 19.9 |
| 4 | *tbl2* | leaf | 502.1 | 101.9 | 87.5 | 20.3 | 17.4 |
| 4 | *tbl2* | leaf | 502.7 | 109.6 | 80.5 | 21.8 | 16.0 |
| 4 | *tbl3* | leaf | 501.3 | 114.3 | 104.5 | 22.8 | 20.8 |
| 4 | *tbl3* | leaf | 500.5 | 110.8 | 94.1 | 22.1 | 18.8 |
| 4 | *tbl3* | leaf | 503.3 | 99.5 | 56 | 19.8 | 11.1 |
| 5 | WT | leaf | 500 | 112.4 | 108.6 | 22.5 | 21.7 |
| 5 | WT | leaf | 507.5 | 121.5 | 125.8 | 23.9 | 24.8 |
| 5 | WT | leaf | 499.6 | 116.1 | 62.8 | 23.2 | 12.6 |
| 5 | *gaut10-2* | leaf | 500.6 | 115.9 | 165 | 23.2 | 33.0 |
| 5 | *gaut10-2* | leaf | 506.6 | 117.3 | 135 | 23.2 | 26.6 |
| 5 | *gaut10-2* | leaf | 503.8 | 123.4 | 150.4 | 24.5 | 29.9 |
| 5 | *gaut11-2* | leaf | 500.9 | 121 | 111.7 | 24.2 | 22.3 |
| 5 | *gaut11-2* | leaf | 503 | 117.7 | 133.1 | 23.4 | 26.5 |
| 5 | *gaut11-2* | leaf | 501 | 125.6 | 95 | 25.1 | 19.0 |
| 5 | *tbr* | leaf | 500.8 | 115.3 | 129 | 23.0 | 25.8 |
| 5 | *tbr* | leaf | 500.3 | 110.2 | 127 | 22.0 | 25.4 |
| 5 | *tbr* | leaf | 501.9 | 119.6 | 132.8 | 23.8 | 26.5 |
| 6 | WT | leaf | 511.7 | 124.4 | 167.9 | 24.3 | 32.8 |
| 6 | WT | leaf | 503.1 | 107.6 | 158.5 | 21.4 | 31.5 |
| 6 | WT | leaf | 502.7 | 121.8 | 151.9 | 24.2 | 30.2 |
| 6 | *axy4-4* | leaf | 503.8 | 127.6 | 140.1 | 25.3 | 27.8 |
| 6 | *axy4-4* | leaf | 503.5 | 114.9 | 156 | 22.8 | 31.0 |
| 6 | *axy4-4* | leaf | 503.8 | 115.1 | 143.8 | 22.8 | 28.5 |
| 6 | *tbl31* | leaf | 508.4 | 102.7 | 140.3 | 20.2 | 27.6 |
| 6 | *tbl31* | leaf | 505.8 | 126.4 | 120.8 | 25.0 | 23.9 |
| 6 | *tbl31* | leaf | 501.8 | 105.2 | 163.6 | 21.0 | 32.6 |

**Supplementary Figure S1. Phenotypes of mutant and wild-type plants at the developmental stages used for tissue collection.** (a) Rosette phenotypes of 3-week-old, pre-bolting plants used for leaf collection from the mannan, pectin, and esterification-related mutants. Two representative plants are shown for each genotype.
(B) Whole-plant phenotypes at the mature bolting stage used for stem collection from the xylan-related mutants. Mature basal internodes of the primary inflorescence stem from xylan mutants were collected for analysis. Scale bar = 5 cm.

**Supplementary Figure S2. Reproducibility of wild-type GPC-ELISA profiles across independent experiments. (a) Overlays of individual biological-replicate Col-0 CDTA-fraction profiles from six independent experiments, comprising three inflorescence-stem experiments (Stem 1–3) and three rosette-leaf experiments (Leaf 1–3). Nine profiles are shown per tissue (three experiments, each with three biological replicates), with experiments distinguished by colour and biological replicates by line style. Profiles are shown for LM15, LM19, LM20, LM21, and LM28. (b) Overlays of individual biological-replicate Col-0 NaOH-fraction profiles for LM15, LM19, LM21, and LM28. LM20 was not analysed in the NaOH fraction because alkaline extraction removes methyl esters.**

Supplementary Figure S3. Statistical analysis of wild-type GPC-ELISA profile reproducibility. (a) Pairwise Pearson correlation coefficients calculated between individual biological-replicate profiles within each tissue type (nine profiles per tissue), across all profiles and within each experiment (three replicates per experiment). The dashed line indicates r = 0.8. (b) Within-tissue cross-experiment coefficients of variation and variation among biological replicates for each antibody–extraction combination. For the biological-replicate CV analysis, technical duplicate ELISA readings were first averaged, and only fractions with detectable signal (mean OD > 0.05) were included. (c) Variance components analysis showing the percentage of total variance attributable to between-experiment and within-experiment (biological replicate) sources for each antibody–extraction–tissue combination, estimated using a one-way random-effects model. Full numerical results are provided in Supplementary Table S2.
